# The darkness and the light: diurnal rodent models for seasonal affective disorder

**DOI:** 10.1242/dmm.047217

**Published:** 2021-01-26

**Authors:** Anusha Shankar, Cory T. Williams

**Affiliations:** Institute of Arctic Biology, University of Alaska Fairbanks, Fairbanks, AK 99775, USA

**Keywords:** Affective behaviors, Photoperiod, Circadian disruption, Depression, Diurnal models, Mood

## Abstract

The development of animal models is a critical step for exploring the underlying pathophysiological mechanisms of major affective disorders and for evaluating potential therapeutic approaches. Although most neuropsychiatric research is performed on nocturnal rodents, differences in how diurnal and nocturnal animals respond to changing photoperiods, combined with a possible link between circadian rhythm disruption and affective disorders, has led to a call for the development of diurnal animal models. The need for diurnal models is most clear for seasonal affective disorder (SAD), a widespread recurrent depressive disorder that is linked to exposure to short photoperiods. Here, we briefly review what is known regarding the etiology of SAD and then examine progress in developing appropriate diurnal rodent models. Although circadian disruption is often invoked as a key contributor to SAD, a mechanistic understanding of how misalignment between endogenous circadian physiology and daily environmental rhythms affects mood is lacking. Diurnal rodents show promise as models of SAD, as changes in affective-like behaviors are induced in response to short photoperiods or dim-light conditions, and symptoms can be ameliorated by brief exposure to intervals of bright light coincident with activity onset. One exciting avenue of research involves the orexinergic system, which regulates functions that are disturbed in SAD, including sleep cycles, the reward system, feeding behavior, monoaminergic neurotransmission and hippocampal neurogenesis. However, although diurnal models make intuitive sense for the study of SAD and are more likely to mimic circadian disruption, their utility is currently hampered by a lack of genomic resources needed for the molecular interrogation of potential mechanisms.

## Abstract (precis)

**Summary:** We review the potential neural mechanisms by which light influences seasonal depression, focusing on research using diurnal rodent models.

## Introduction

Seasonal affective disorder (SAD) is diagnosed based on a seasonally recurring pattern of depression that typically coincides with short photoperiods in fall or winter and subsequently abates each spring or summer (Sandman et al., 2016; Sohn and Lam, 2005). In addition to depression, a significant subset of SAD patients display a multitude of ‘atypical’ secondary symptoms, including sleep disruption, fatigue, carbohydrate craving and weight gain (Young et al., 1991). The syndrome was first described by Rosenthal et al. (1984) and has subsequently been entered in several editions of the Diagnostic and Statistical Manual of Mental Disorders, including the most recent edition (DSM-5; American Psychiatric Association, 2013). The prevalence of SAD varies markedly between the sexes, with women being three to five times more likely to suffer than men (Magnusson, 2000; Wirz-Justice et al., 2019).

Prevalence estimates for SAD have ranged between 1.4% and 9.7% in North America, 1.3% and 3.0% in Europe, and 0% and 0.9% in Asia (reviewed in Magnusson, 2000). Further, whereas some studies have reported a dramatic latitudinal cline in prevalence (Kegel et al., 2009; Rosen et al., 1990), others have failed to detect any effect of latitude (Magnusson, 2000; Sandman et al., 2016). The widespread discrepancy in reported prevalence is likely related to variability in the application of diagnostic criteria (Cléry-Melin et al., 2018) and genetic and/or cultural differences among populations (Saheer et al., 2013; Suhail and Cochrane, 1997). There is also a clear need for longitudinal studies that permit the tracking of depression and atypical symptoms of SAD in individuals across time. In one of the few studies to take a longitudinal approach, Wirz-Justice et al. (2019) estimated a prevalence rate of 3.4% in Zurich, Switzerland, with occurrence being five times higher in women than men. Irrespective of the uncertainty regarding prevalence rates, the recurrent course and long episode duration of SAD make it a significant mental health issue, particularly among women.

The development of animal models is a critical step in exploring the underlying pathophysiological mechanisms of this disorder and evaluating potential therapeutic approaches. A cornerstone of biomedical research using animal models is that the results from research on the model system reflect the human disease of interest. This may not be the case for nocturnal mouse and rat models of SAD, given that SAD is linked to circadian disruption, and that the effects of day length on behavioral rhythms differ between diurnal and nocturnal species. This mismatch has led to a call for the development of diurnal rodent models to investigate the molecular and neurological mechanisms that link circadian and sleep dysfunction, carbohydrate craving and weight gain, as well as seasonal depression in SAD (Bilu et al., 2016; Workman and Nelson, 2011). Additionally, a major limitation of many studies using rodent models is their tendency to only use males for experiments (reviewed in Workman and Nelson, 2011), which is particularly unfortunate for studies of SAD, because women are at far greater risk.

In this Review, we describe what is known regarding the etiology of SAD, focusing on evidence supporting the purported role of circadian disruption. We then provide a brief overview of how circadian systems function in mammals, focusing on the current state of knowledge regarding the role of circadian disruption in SAD. We argue that although circadian disruption is often cited as the primary mechanism underlying SAD, too many studies fail to provide explicit hypotheses regarding what aspect of circadian physiology is being disrupted and how this is leading to changes in mood, sleep and appetite. We then discuss progress in developing diurnal rodent models to interrogate potential mechanisms. We focus on two diurnal rodent models, the fat sand rat (*Psammomys obesus*) and the Nile grass rat (*Arvicanthis niloticus*), and summarize findings from one avenue of research that holds particular promise – the orexinergic system. Finally, we briefly outline promising future research directions and argue that greater investment is needed in the development of genomic resources for diurnal rodent models to better understand the role that light plays in human mental health.

## The etiology of depression in SAD patients

The monoamine hypothesis, which postulates that concentrations of monoamines – such as serotonin, noradrenaline and dopamine – are decreased in synaptic gaps in the depressive state, was the most commonly accepted hypothesis of major depressive disorder for a long period (reviewed in Hirschfeld, 2000). Serotonin transporter (SERT, the protein encoded by the gene *SLC6A4*) is a protein that transports serotonin from the synaptic cleft to the presynaptic terminal; it terminates the effects of serotonin and allows for its reuse by the neuron. SERT function has repeatedly been found to be enhanced during depression in subjects with SAD (Mc Mahon et al., 2016; Tyrer et al., 2016a; Tyrer et al., 2016b; Willeit et al., 2008). SAD patients had, on average, 5% higher levels of SERT in the winter compared with the summer, corresponding with lower levels of active serotonin, whereas healthy participants showed no significant change (Haahr et al., 2014; Mc Mahon et al., 2018). Selective serotonin reuptake inhibitors (SSRIs), which function by blocking the reuptake of serotonin into nerve terminals through SERT, are among the most commonly used antidepressants for major depressive disorders. SSRIs are also used to treat SAD and appear to be effective when used alone or in combination with bright-light (BL) treatment (e.g. Blashko, 1995; Lam et al., 2006, 2016; Pjrek et al., 2009). However, trials on SAD patients have generally been small, suffer from issues with study design and have high drop-out rates, owing to adverse side effects of SSRIs (reviewed in Thaler et al., 2011).

An important issue with the monoamine hypothesis is that SSRIs act to restore monoamine levels within hours, but the beneficial effects on mood appear only after weeks of treatment (Taylor et al., 2006). It has now been shown that the stimulation of neurogenesis by antidepressants contributes to their behavioral effects (Malberg et al., 2000; Santarelli et al., 2003), and it has been proposed that a stress-induced decrease in neurogenesis in the dentate gyrus is an important causal factor in precipitating episodes of depression (Snyder et al., 2011). Thus, SSRIs may be ameliorating depression by elevating serotonin concentration at synaptic terminals of serotonergic neurons projected into the dentate gyrus, thereby directly increasing the proliferation of neural precursor cells (reviewed in Boku et al., 2018). Alternatively, stress might induce the atrophy of hippocampal neurons (i.e. a shortening of dendrites and a decrease in the density of spines), which is slowly reversed by antidepressants (Watanabe et al., 1992). This has led to increased interest in the development of novel antidepressants that rapidly induce synaptogenesis and spine formation (Duman and Li, 2012).

## The role of light

**Fig. 1.**
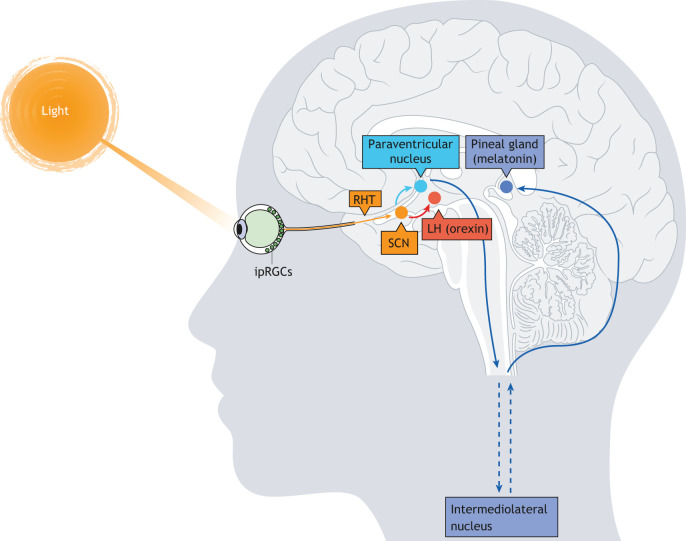
**Pathways through which light might influence mood and reward systems in mammals.** (A) The retinohypothalamic tract (RHT), in which axons from intrinsically photosensitive retinal ganglion cells (ipRGCs) project directly to the central clock – the suprachiasmatic nucleus (SCN). The SCN influences the secretion of melatonin by the pineal gland through the paraventricular nucleus and the intermediolateral nucleus. It also influences the orexin system via projections to the lateral hypothalamus (LH). The geniculohypothalamic tract, which connects ipRGCs to the SCN via the intergeniculate leaflet in the thalamus, is not shown.

Given that depression and other atypical depressive symptoms of SAD vary seasonally, with the severity of symptoms coinciding with short photoperiods, light is presumed to play a crucial role in the disorder (American Psychiatric Association, 2013). In mammals, intrinsically photosensitive retinal ganglion cells (ipRGCs) convey light information to a variety of post-synaptic targets, principally including the suprachiasmatic nucleus (SCN) (Fig. 1; LeGates et al., 2014). The molecular circadian clockwork within each cell of the SCN allows it to remain rhythmic with a period of ∼24 h, even in the absence of environmental cues (Herzog et al., 2017). The SCN (the ‘central clock’), in turn, acts to synchronize cell-autonomous circadian oscillators found in other brain regions and the periphery, including non-neuronal tissues (‘peripheral clocks’), through synaptic connections and/or by driving circadian rhythms in body temperature and hormone production and release (Buhr et al., 2010; Pfeffer et al., 2018; Weger et al., 2016). The SCN also innervates the pineal gland, which releases melatonin during the scotophase (dark phase), by a multi-synaptic pathway via the paraventricular nucleus (see Fig. 1). Melatonin plays a role in entraining peripheral clocks to the central clock (Pevet and Challet, 2011) and has been associated with mood disorders, including bipolar disorder and SAD. SAD patients seem to exhibit higher melatonin levels and phase-delayed melatonin onset in the winter, compared with healthy controls (Danilenko et al., 1994; De Berardis et al., 2015; Lewy et al., 1998; Srinivasan et al., 2006; Wehr et al., 2001).

Disruption of circadian rhythms has long been hypothesized to underlie the development of mood disorders, including SAD (McClung, 2007; Vadnie and McClung, 2017). Circadian disruption can include misalignment between daily environmental rhythms and the SCN (Lewy et al., 2007), a lack of synchrony between pacemaker cells within the SCN (Ben-Hamo et al., 2016b), misalignment between the SCN and peripheral clocks (Mohawk et al., 2012), and/or the abolishment or dampening of the rhythmic expression of clock and/or clock-controlled genes (Musiek, 2015). The phase-shift hypothesis (PSH) proposes that depressive episodes are caused by misalignments (usually a phase delay) in circadian rhythms relative to the sleep-wake cycle (Lewy et al., 1988, 2006, 2007). This hypothesis is tightly linked with two therapeutic treatments for SAD that do not involve the use of antidepressants to modulate brain monoamines: treatment with BL, typically in the morning (Campbell et al., 2017), and treatment with melatonin, typically in the early evening (Rosenthal et al., 1986). Although widely used, empirical support for the effectiveness of these treatments remains controversial. In a recent meta-analysis, Nussbaumer-Streit et al. (2019) found that the efficacy and safety of melatonin for prevention of SAD cannot be conclusively supported, owing to a lack of controlled clinical trials. Likewise, Pjrek et al. (2020) remark that evidence for BL therapy comes from methodologically heterogeneous studies with small-to-medium sample sizes, but conclude that BL therapy is effective. Others take issue with the lack of effective controls in BL therapy studies (Golden et al., 2005), and larger high-quality clinical trials are clearly needed. Although it is generally presumed that BL treatment, if effective, exerts its effects by reducing circadian disruption, recent studies suggest that light can directly affect mood without the involvement of the SCN (reviewed in LeGates et al., 2014), creating the possibility that these therapeutic effects may be circadian independent (see below).

## The genetics of SAD

Similar to schizophrenia, bipolar disorder and major depressive disorders, SAD is thought to be polygenic (Byrne et al., 2015; Partonen, 2012), meaning that many small genetic risk factors influence risk in the population and that no gene or variant on its own is likely to be fully deterministic. Using a candidate gene approach (i.e. investigations of specific genes based on *a priori* knowledge of their function), Rosenthal et al. (1998) initially found that the short allele of the SERT promoter repeat length polymorphism contributes to the trait of seasonality and is a risk factor for SAD, although follow-up studies have provided mixed support for this result (reviewed in Garbazza and Benedetti, 2018). Single-nucleotide polymorphisms in clock genes encoding neuronal PAS domain-containing protein 2 (NPAS2), period circadian protein homolog 2 (PER2), circadian locomotor output cycles protein kaput (CLOCK) and aryl hydrocarbon receptor nuclear translocator-like protein 1 (ARNTL), and in the gene encoding melanopsin (OPN4), have also been linked to SAD using the targeted gene approach (Johansson et al., 2003; Kim et al., 2015; Partonen et al., 2007; Roecklein et al., 2009). Ho et al. (2018) conducted a genome-wide association study and identified an intronic variant (rs139459337) in *ZBTB20*, which encodes a transcriptional repressor that has roles in neurogenesis (Xie et al., 2010), as the strongest candidate gene for susceptibility to SAD. In nocturnal laboratory mice, knockout of *Zbtb20* alters circadian rhythms of behavior and impairs their ability to entrain to a shortened day (Qu et al., 2016). Thus, the limited results available from human genetic studies appear to provide some support for the circadian disruption hypothesis.

## A role for orexin?

**Fig. 2.**
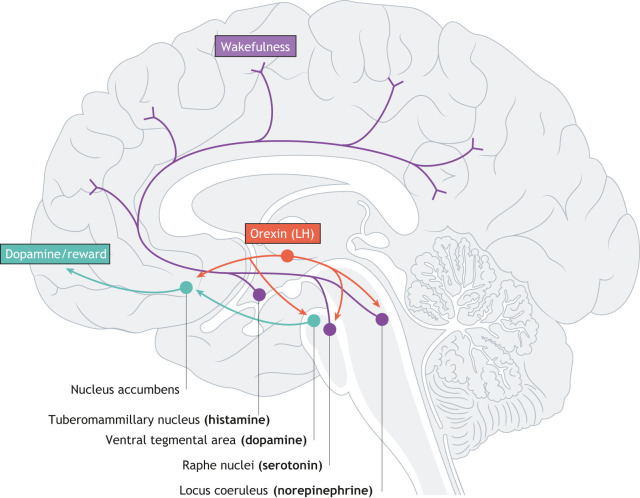
**The main projections of orexin neurons.** Orexin has direct effects on sleep and wakefulness, and possibly mood. Additionally, the orexin system may have indirect effects on symptoms of SAD; orexin affects dopamine and serotonin, which influence reward-seeking behavior and mood. LH, lateral hypothalamus. Redrawn from BaHammam et al. (2015). This image is not published under the terms of the CC-BY license of this article. For permission to reuse, please see BaHammam et al. (2015).

Orexin (also known as hypocretin) neuropeptides regulate several homeostatic functions, including the sleep/wake cycle, food intake, energy homeostasis and arousal (reviewed in Tsujino and Sakurai, 2009). Orexin neurons are concentrated in the perifornical area of the lateral hypothalamus (LH) across vertebrates (Johnson et al., 1988) and project to and activate much of the central nervous system (Fig. 2). Dysregulation of orexin has severe consequences: loss of orexin neurons is the most common cause of narcolepsy in humans, a disease characterized by excessive daytime sleepiness (Scammell, 2015). Narcolepsy is also frequently comorbid with mood and anxiety disorders (Fortuyn et al., 2010; Vourdas et al., 2002). Although the mechanisms through which orexins influence mood remain unclear, orexin neurons can regulate serotonin directly, by exciting serotonergic neurons in the dorsal raphe nuclei, which abundantly express both orexin receptors (OX1R and OX2R, encoded by genes *HCRTR1* and *HCRTR2*, respectively), or indirectly, by inhibiting local GABAergic inputs to serotonergic neurons (Liu et al., 2002). The orexinergic system also modulates the norepinephrine system, and a network involving ipRGCs, the SCN, orexin and norepinephrine has been proposed as being involved in depressive disorders, including SAD (Bowrey et al., 2017).

**Fig. 3.**
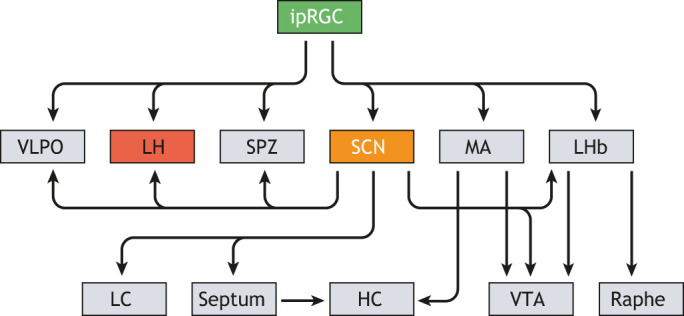
**Direct projections from ipRGCs to numerous brain regions, including the SCN.** Many of the ipRGC (green) targets also receive innervation from the SCN (orange) and it is possible that, in addition to its role as a pacemaker, the SCN acts as a conduit for light information. Two regions – the medial amygdala (MA) and the lateral habenula (LHb) – also act as brain peripheral clocks that receive direct retinal innervation. The lateral hypothalamus (LH; red) is the region with the greatest density of orexin neurons, and it can receive light information directly from the ipRGCs, as well as from the SCN. Orexin systems are also likely sensitive to melatonin production (not shown). The ventral tegmental area (VTA) and raphe (the primary site of serotonin secretion), both of which are areas involved in mood regulation, as well as the hippocampus (HC), involved in cognition, can be influenced by the effects of light on ipRGCs that connect to the SCN or by ipRGCs that connect to the MA and/or LHb (projections from raphe to HC not shown). LC, locus coeruleus; SPZ, subparaventricular zone; VLPO, ventrolateral preoptic area. Redrawn from LeGates et al. (2014). This image is not published under the terms of the CC-BY license of this article. For permission to reuse, please see LeGates et al. (2014).

The potential for disruptions in orexigenic signals to be involved in SAD stems from evidence that the hypothalamic orexin system plays a key role in modulating the effects of light on the monoaminergic systems and influences appetite, food cravings, reward pathways and sleep cycles (Cao and Guilleminault, 2011; Cason et al., 2010; Harris et al., 2005; Sakurai et al., 2010), all of which are disturbed in SAD patients (Levitan, 2007; Tsujino and Sakurai, 2013; Tsuneki et al., 2016). For example, orexin gene expression in the LH of sheep is influenced by photoperiod: orexin gene expression is higher in short photoperiods [8 h:16 h light:dark (LD)], relative to long photoperiods (16 h:8 h LD, Archer et al., 2002; Zieba et al., 2011). The effects of light on the orexin system can be indirect, and attributable to effects on the SCN, or direct, occurring through innervation of ipRGCs to other brain regions, including the LH (Fig. 3; Hattar et al., 2006; LeGates et al., 2014; Marston et al., 2008). The indirect effects of light on orexin are mediated either by changes in circadian rhythms within the SCN, which controls orexin neuron activation, or by the effects of the SCN on melatonin released by the pineal gland (Fig. 1). Orexin neurons in the perifornical region of the LH in mice express melatonin type 1 receptors, which likely contribute to the effects of melatonin upon the sleep-wake cycle (Sharma et al., 2018). Conversely, orexin can influence circadian rhythmicity by affecting melatonin production. Orexin directly affects melatonin synthesis in rats (Mikkelsen et al., 2001), and orexins have been found to modulate melatonin production at night in zebrafish (Appelbaum et al., 2009). The existence of a direct neural pathway between light and the LH, the location of the central hub of the orexinergic system, has given rise to the possibility that photoperiod and/or dim light can alter the orexinergic system and induce affective behaviors independent of the circadian clock (see below).

## Assessing affective behaviors in animal models

One difficulty in using rodent models to assess mental health is developing reliable criteria for assessing mood. Although several tests have been developed to assess changes in aspects of affective-like behaviors in rodents, including tests for depression-like behaviors, anxiety-like behaviors and symptoms such as anhedonia, the validity of these approaches is debated, and the application of these methods varies among research groups (Bogdanova et al., 2013; Kara et al., 2018). The most commonly used assay is the forced swim test (FST; Box 1), which remains the standard method for assessing changes in antidepressant activity (Yankelevitch-Yahav et al., 2015), and it is often used within studies as an indicator of changes in affective-like behavior in rodent models. Although we note that behavior in the FST can be influenced by factors unrelated to depression (such as general activity levels, age, handling, diet; Bogdanova et al., 2013), for the remainder of this Review, we consider time to sink or immobility times in the FST to be an adequate within-study proxy for changes in affective behavior.

**Box 1. Various tests used to assess changes in depressive-like behavior in rodents.**

**Forced** **swim** **test** **(FST)**: the FST involves measuring the time a rodent spends immobile, not trying to escape, from a cylindrical swim chamber (Yankelevitch-Yahav et al., 2015). It is now often used to detect changes in depression-like behavior. The classic viewpoint is that floating, rather than attempting to escape, indicates despair, and that immobility time can be used as a proxy for depressive-like behavior: a shorter ‘give-up” time indicates a more depressed individual (Cryan and Slattery, 2012). However, other interpretations have been proposed, including that a more rapid transition from swimming to floating indicates a stress-coping strategy (Molendijk and de Kloet, 2019) or that continuous swimming is indicative of a panic or anxiety response (Anyan and Amir, 2018). There is also variation between species in how the FST is administered; the measure used to assess changes in affective-like behaviors in the FST for fat sand rats is time to sink, whereas for Nile grass rats and many other rodents it is immobility time (Ashkenazy-Frolinger et al., 2010; Leach et al., 2013a; Porsolt et al., 1978; Tal-Krivisky et al., 2015; Workman and Nelson, 2011). Conversely, the FST has been proven successful in evaluating the effectiveness of antidepressant drugs in nocturnal rodent models (Kara et al., 2018; Unal and Canbeyli, 2019), and performance in the FST is correlated with altered serotonin transporter (SERT) expression (Ulloa et al., 2014). Thus, the FST remains the standard method for assessing changes in antidepressant activity, and it is often used within studies as an indicator of changes in affective-like behavior in rodent models.

**Sweet-solution** **preference test** **(SSP)**: in the SSP, a reduction in the preference ratio for sweet solution (either saccharine or sucrose) over water in experimental versus control animals is considered indicative of anhedonia. Anhedonia involves a dysregulation in the reward circuit, and manifests as a diminished interest or pleasure in all or almost all activities. Although anhedonia in the SSP might seem inconsistent with carbohydrate craving – a well described symptom of SAD – this could be due to the low concentration of sucrose used in tests (1–3%). Although rodents exhibit anhedonia when presented with this lower-concentration sucrose solution in the SSP (i.e. their reward pathways are not activated at low concentrations), they typically increase sucrose solution consumption when presented with a high-concentration (10%) sucrose solution (Sinitskaya et al., 2008; Workman and Nelson, 2011). In the case of diurnal rodent models, there appears to be some variability in the application of the SSP – e.g. in the number of days of acclimation to the sweet solution bottle – or whether saccharin or sucrose are administered (Ashkenazy-Frolinger et al., 2015; Ashkenazy et al., 2009b; Leach et al., 2013a).

The sweet solution preference test (SSP; Box 1) is often used to assess anhedonia (a diminished interest or pleasure in all or almost all activities), which represents a dysregulation of the reward circuit and is associated with depressive disorders (Papp et al., 1991). Similar to the FST, the SSP sometimes produces inconsistent results among researchers owing to variability in the protocols and equipment used (Liu et al., 2018). Although the FST and SSP are the most pertinent tests when it comes to SAD, there are a variety of other commonly used tests for anxiety-like behaviors, including open-field tests, light/dark box tests and elevated plus maze tests (see Box 2; Harro, 2018; Walf and Frye, 2007). These tests are increasingly being used to understand whether there is comorbidity between anxiety and depression in rodents, given that this comorbidity often occurs in humans.

**Box 2. Tests used to assess changes in anxiety-like or anxiogenic behaviors in rodents.**

**Elevated plus maze test**: this test measures how rodents respond to a novel approach or avoidance situation. The elevated plus maze usually consists of two open arms and two closed arms with walls and an open roof, with the similar arms opposite from each other. The maze is elevated to an approximate height of 0.5 m. For testing, the animal is placed in the center of the maze, and scored on how many times it enters the open versus closed arms, and on the time it spends in open versus closed arms. Animals that spend relatively more time in the enclosed arms and cross between arms infrequently are considered as showing greater anxiogenic behavior (File, 1993; Leach et al., 2013a).

**Light/dark box test**: this test for changes in anxiety-like behaviors was originally developed in male mice (Hascoët et al., 2001; Onaivi and Martin, 1989) and was based both on the aversion of nocturnal rodents to brightly lit areas, and on their spontaneous exploratory behavior. The light/dark box is divided into two compartments: a black, covered, ‘dark’ compartment (one-third of the box) and a white, uncovered ‘light’ compartment (two-thirds of the box), with a separating door that allows the animal to move between compartments, usually for a 5-min session (Ashkenazy-Frolinger et al., 2015; Hascoët et al., 2001). Time spent in the light versus dark regions of the box is used as a measure of changes in anxiety-like behavior.

**Open-field test**: the open field comprises a walled arena, often marked with a grid and square crossings. The center of the field is marked with a different color to differentiate it from the other squares, and changes in anxiety-like behavior are assessed by a combination of frequency of movement across squares and other behaviors (Gould et al., 2009; Leach et al., 2013a).

**Social interaction and aggression** **tests**: in these tests, the animal is first placed alone in an arena, and the baseline amount of time that it spends in a zone that will later contain a social interaction partner is measured. Once a social partner is introduced into that area of the arena, changes in anxiety-like behavior are measured by the time the target individual spends with the partner, and the ratio of the time spent in the zone with and without the social partner. Aggressive behaviors such as fighting and attempts at biting and pinning down are also recorded. (File and Seth, 2003; Lezak et al., 2017; Tal-Krivisky et al., 2015).

## The need for diurnal models

In the case of SAD and other mood disorders that are influenced by light, the validity of using nocturnal models has been questioned for a number of reasons (Bilu et al., 2016; Workman and Nelson, 2011; Yan et al., 2019). For example, the mechanisms that determine the active phases of the circadian system of nocturnal and diurnal animals are fundamentally different, as are their responses to changes in day length (Bilu et al., 2016). Additionally, arousal-dependent non-photic stimuli provide synchronizing feedback signals to the SCN in circadian antiphase between nocturnal and diurnal animals (Challet, 2007). Human responses to changes in day length, or light intensity, might therefore be better reflected in the way diurnal animal models respond to light. For example, when exposed to short photoperiods, nocturnal animals experience a lengthened active phase, whereas diurnal animals typically experience a compressed active phase (Refinetti, 2004). All strains of nocturnal laboratory mice, except one, show no clear behavioral changes under short, neutral or reversed (19 h:5 h LD) photoperiods, demonstrating that diurnal rodents are not simply a mirror image of nocturnal rodents (Bilu et al., 2019a; Flaisher-Grinberg et al., 2011; Otsuka et al., 2014; Roseboom et al., 1998). In humans, the number of dopaminergic neurons in the midbrain increases in response to long photoperiods (Aumann et al., 2016), whereas in nocturnal rats the number of hypothalamic dopaminergic neurons decreases under long photoperiod exposure (Dulcis et al., 2013).

Light entrains or resets the circadian clock of the SCN very similarly in nocturnal and diurnal mammals, but circadian rhythms in other brain regions or in the periphery are influenced by temporal niche (i.e. nocturnality or diurnality; Challet, 2007; Jha et al., 2015). Additionally, the circadian-independent direct effects of light on the brain and behavior are very different between diurnal and nocturnal species (Smale et al., 2003; Yan et al., 2018, 2019). For instance, the orexin neurons of nocturnal laboratory rats are activated at night (Estabrooke et al., 2001) by darkness (Marston et al., 2008; Mendoza et al., 2010), whereas orexin signaling is highest during the day (Smale et al., 2003) and activated by light in diurnal grass rats (Adidharma et al., 2012). This difference could be mediated by differences in the relative proportions of the various ipRGC types in the retinas of diurnal versus nocturnal rodents. One component of this difference is that M1-type ipRGCs constitute 74% of all ipRGCs in a diurnal rodent species, the Sudanian grass rat (*Arvicanthis ansorgei*), compared with 30–44% in the mouse retina (Karnas et al., 2013). This higher proportion of M1 cells in the diurnal rodent, together with their higher light sensitivity than those in mice, might contribute to their ipRGCs mediating a different circadian behavior than they would in nocturnal mice (Karnas et al., 2013). Further, activation of γ-aminobutyric acid type A (GABA_A_) receptors by a GABA_A_ agonist produces phase advances in the SCN of nocturnal rodents, but causes phase delays in diurnal rodents, even when administered at the same circadian times (Novak and Albers, 2004; Smith et al., 1989).

However, although there is a clear rationale for developing diurnal models for SAD, and potentially for other psychiatric disorders (Lam et al., 2016), not all diurnal animals will necessarily be appropriate. Fat sand rats, Nile grass rats, Sudanian grass rats, Golden spiny mice (*Acomys russatus*), Degus (*Octodon degus*), tuco-tucos (*Ctenomys aff. knighti*), Mongolian gerbils (*Meriones unguiculatus*) and Syrian hamsters (*Mesocricetus auratus*) have all been considered as diurnal model systems (Ashkenazy-Frolinger et al., 2015; Ben-Hamo et al., 2016a; Bilu et al., 2016; Tomotani et al., 2012; Yan et al., 2018). Of these models, the fat sand rat and Nile grass rat have received the most attention in the study of SAD. Workman and Nelson (2011) have suggested that species such as the Syrian hamster, which seasonally fattens in response to short photoperiods, could be good models for SAD, because this fattening is consistent with disrupted energy homeostasis. However, hyperphagia in these animals is an adaptive response to decreasing day length in early fall, rather than reflecting a chronic condition during the winter months, because prolonged exposure to short days results in decreased food intake and reductions in body mass (Wade and Bartness, 1984). We contend that species that evolved in regions that are less seasonally variable in photoperiod, and are thus experiencing photoperiodic conditions in the laboratory or in semi-natural conditions under which they did not evolve (Bilu et al., 2019a), are better suited as models of SAD (i.e. they may better reflect SAD in humans that have migrated away from their more equatorial origins).

One major limitation of many diurnal rodent models is the propensity to shift to a more nocturnal phenotype under laboratory conditions. For example, although the fat sand rat and Nile grass rat have both been promoted as models of SAD, neither species is exclusively diurnal under laboratory conditions; the fat sand rat becomes nocturnal when housed in the laboratory (Barak and Kronfeld-Schor, 2013), whereas some, but not all, grass rats become nocturnal when housed in captivity with a running wheel (Blanchong et al., 1999). To alleviate this issue, studies using grass rats for SAD research typically do not provide animals with running wheels. Rodent models, in general, exhibit far more circadian plasticity than humans. This greater plasticity could be due to their small body size and need to balance energy budgets on relatively short time-scales, such that they must rapidly shift to more diurnal behavior when food availability is reduced (Riede et al., 2017).

## Progress made using diurnal models

As a first step towards developing diurnal rodents as models of SAD, researchers exposed animals to short photoperiods and employed behavioral assays to assess the impact of short photoperiods on depression, anhedonia, anxiety and cognition. For example, when exposed to short photoperiods, sand rats exhibit depression, anhedonia and anxiety, as assessed using the FST, SSP and elevated plus maze test, respectively (Ashkenazy et al., 2009a; Einat et al., 2006). Melatonin administered in a fashion to mimic a long night signal (short photoperiod) also induces depression and anxiety-like phenotypes (Ashkenazy et al., 2009a). Treating sand rats with the antidepressant bupropion, a norepinephrine-dopamine reuptake inhibitor, reversed the depressive effects of short photoperiod (Krivisky et al., 2011). Additionally, BL treatment for 1 h at the onset of ‘lights on’ (i.e. at the start of the light phase) ameliorated symptoms of depression and anxiety, but not anhedonia (Ashkenazy et al., 2009b). Interestingly, blue light was as effective as wide-spectrum BL (Bilu et al., 2019b). Fat sand rats maintained in short photoperiods lost their rhythm of *Per2* mRNA expression, whereas those under neutral photoperiod maintained it (Bilu et al., 2019a). Providing a running wheel for voluntary exercise also strengthened the circadian organization of general activity and reduced depression and anxiety (Tal-Krivisky et al., 2015). Thus, evidence from sand rats appears consistent with some form of circadian disruption being implicated in SAD.

Other species of diurnal rodents show similar responses to short photoperiods; for example, Degus exhibit depression-like behavior and anxiety, based on the FST and open-field test, respectively (Ashkenazy-Frolinger et al., 2015). The Mongolian gerbil exhibits depressive-like and anxiety-like behavior under short photoperiods, and its diel activity rhythms are almost completely abolished under these conditions (Juárez-Tapia et al., 2015). Maintaining Mongolian gerbils in complete darkness also induces depression-like behavior and suppresses hippocampal neurogenesis (Lau et al., 2011). Studies in the Sudanian grass rat reveal that exposure to short photoperiods depresses the diel amplitude of expression of *Per2*, but not *Arntl* (which is a candidate gene for susceptibility to hypertension, diabetes and obesity, and in which mutations have been linked with altered sleep patterns; Pappa et al., 2013; Richards et al., 2014) in the SCN. Diel variation in dopaminergic neurotransmission in the nucleus accumbens and the dorsal striatum was also affected (dopamine in these regions signals feeding and other reward- and goal-directed behaviors; Floresco, 2015; Itzhacki et al., 2018; Palmiter, 2008). Interestingly, whereas dopamine disruption was reversed in animals exposed to BL treatment early or late in the day, the phase of the daily rhythm of locomotion reverted only in animals exposed to BL late in the day (Itzhacki et al., 2018). This is consistent with the timing of dusk being a more important zeitgeber (environmental cue that entrains an organism's circadian clock) than dawn (Challet, 2007).

**Fig. 4.**
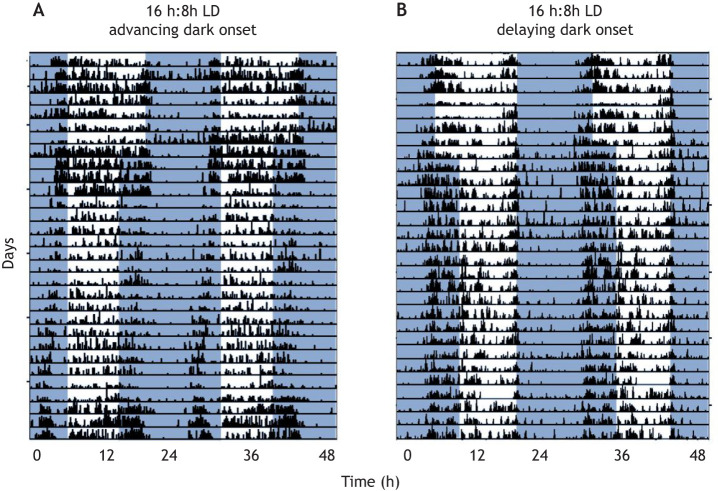
**Activity changes in Nile grass rats upon short-photoperiod treatment.** (A,B) Double-plotted actograms of Nile grass rat (*Arvicanthis niloticus*) individuals housed initially in neutral photoperiod [12 h:12 h light:dark (LD)] and then shifted to a shorter photoperiod (16 h:8 h LD), either by advancing dark onset (A) or delaying light onset (B). To better visualize activity rhythms, actograms are often double plotted by placing data from two consecutive days horizontally (e.g. Day 1 and 2 in the first row; Day 2 and 3 in the second). White portions of the plot represent the light phase and blue portions represent the dark phase. The *y*-axis shows time in these overlapping 48 h intervals from top to bottom. The *x*-axis shows time in hours. The black bars show activity measured with motion sensors, with higher bars representing greater activity. Reproduced from Leach et al. (2013b). This image is not published under the terms of the CC-BY license of this article. For permission to reuse, please see Leach et al. (2013b).

In contrast to sand rats and Sudanian grass rats, the circadian system of Nile grass rats does not appear to be strongly disrupted in response to short-photoperiod treatments. The timing of their activity rhythms remains largely synchronous between animals on short and long photoperiods, with activity onset occurring ∼12 h before the transition from light to dark, although activity is reduced in the early part of the subjective day, when it remains dark (Leach et al., 2013b; Fig. 4). Further, there was no evidence for a difference between short (8 h:16 h LD) and neutral (12 h:12 h LD) photoperiod groups in the amplitude or timing of PER1 and PER2 protein expression in the SCN (Leach et al., 2013b). However, Nile grass rats exposed to short photoperiods do exhibit depressive-like behaviors and anhedonia; Leach et al. (2013b) propose that the inability of these equatorial rodents to alter their circadian rhythms and reduce the duration of their daily active phase may make them more vulnerable to the effects of short photoperiod on mood.

Most humans have access to artificial light, which is much dimmer than sunlight, but can act to extend the duration of the natural photoperiod in winter. As such, it has been argued that SAD can be triggered by exposure to dim light, in addition to exposure to a short photoperiod (Leach et al., 2013b). Dim-light treatments have therefore been frequently used over the past decade to assess SAD. An added benefit of using a neutral photoperiod dim-light treatment is that it allows one to ensure that sampling of treatment and control groups occurs at the same phase of the circadian clock. When Nile grass rats are exposed to a neutral dim [12 h:12 h dim light:dark (DLD)] versus neutral bright photoperiods [12 h:12 h BL:dark (BLD)], circadian rhythms seem unaffected, with the total daily activity, day/night activity ratio and entrainment phase angle (i.e. the relationship between the timing of the biological clock and the timing of an external time cue) all remaining largely consistent between bright and dim-light treatments, even though these animals exhibit symptoms of depression under the 12 h:12 h DLD treatment based on the FST and SSP (Leach et al., 2013a). To study which neural pathways are involved in mediating the effects of light on mood regulation, Adhidharma et al. (2012) maintained Nile grass rats in constant darkness and exposed them to BL early in their subjective day. They found that treatment with a light pulse did not increase neural activity in the SCN, but did increase the activity of orexin neurons innervating the dorsal raphe nucleus (DRN), as determined by *Fos* expression (Adidharma et al., 2012). Based on these findings and the direct pathway between ipRGCs and the LH (Fig. 3), it has been proposed that short photoperiods or dim-light conditions can induce affective-like behaviors through the direct effects of light on the hypothalamic orexin system (Yan et al., 2019).

Because orexin neurons project into the DRN, among other central monoaminergic systems (Liu et al., 2002), they could potentially downregulate the serotonin system and affect mood and anxiety. Increased depression- and anxiety-like behaviors were correlated with the attenuation of orexin fibers and a decrease in the number of serotonin neurons in the DRN of Nile grass rats, along with a lower density of serotonin fibers/terminals in the anterior cingulate cortex (Deats et al., 2014; Leach et al., 2013a). Both dim-light (12 h:12 h DLD) and short-photoperiod (8 h:16 h BLD) treatments lead to a decrease in the number of hypothalamic dopaminergic and inhibitory somatostatin neurons (Deats et al., 2015). Of the two orexin peptides (orexin A and orexin B) and the orexin receptors (OX1R and OX2R), orexin A and OX1R seem to play the primary roles in influencing affective-like behaviors (Adidharma et al., 2012; Adidharma et al., 2019). Infusing orexin A into the DRN increases local extracellular serotonin by 200–300%, whereas infusing orexin B, even at a higher dose, causes only a 20–30% increase (Adidharma et al., 2019). Even under 12 h:12 h BLD conditions, inhibiting orexinergic pathways can induce changes in affective-like behaviors in Nile grass rats. Treating animals in BL conditions (12 h:12 h BLD) with a selective OX1R antagonist (SB-334867, which has 50-fold higher sensitivity for OX1R than for OX2R; Porter et al., 2001; Smart et al., 2001) decreases the number of orexinergic neurons in the hypothalamus, decreases overall neural activity in the DRN and increases their depression-like behaviors (Adidharma et al., 2012; Deats et al., 2014). Although direct retinal innervation of orexinergic neurons has not been shown, retinal innervation of the LH by ipRGCs has been demonstrated in both laboratory rats and Nile grass rats (Gaillard et al., 2013; Leak and Moore, 1997). Orexin peptides and receptors have been found in other brain regions, but their roles in these other brain regions have not been well studied in diurnal rodents. OX1R in Nile grass rats has been more extensively studied because it is localized in the region of the DRN associated with affective behaviors (Adidharma et al., 2019). The role of OX2R in diurnal rodents is not well known; in nocturnal mice, OX2R seems to have antidepressive effects, whereas OX1R is associated with pro-depressive and anxiety-inducing actions (Summers et al., 2020). Further, although the distribution of OX1R and OX2R is broadly similar between diurnal and nocturnal rodents, there are some distinct differences (Ikeno and Yan, 2018); the implications of these differences is currently unclear. Finally, although evidence suggests that dim light results in a reduction in the number of orexin A neurons in the hypothalamus and attenuated orexin A fiber density in the DRN (Deats et al., 2014), and low orexin levels are associated with depressed mood (James et al., 2017), it remains unclear how this melds with hyperphagia in SAD, which presumably involves heightened orexin signaling (Mahler et al., 2014).

## Conclusions and future directions

There is a clear rationale for the development of diurnal animal models for affective disorders, including SAD, and results from studies to date are encouraging in terms of the potential utility of these models. Regarding the etiology of SAD, circadian disruption appears to be a well-supported phenomenon, although what aspects of circadian physiology are disrupted is not entirely clear and recent studies in Nile grass rats suggest that direct, circadian-independent effects of light may also be important. Further, although recent studies using grass rats highlight the potential involvement of the orexinergic system, more work needs to be done to better understand whether these systems are directly influencing mood, or inducing affective behaviors through effects on monoamine systems, neurogenesis and/or neuroplasticity. Recent studies also suggest that the intestinal microbiome might play a previously underappreciated role in neuropsychiatric disorders (Foster and McVey Neufeld, 2013), and although we do not cover this line of research in this Review, we suggest that studies of how the microbiota-gut-brain axis is affected by light in diurnal models could be a profitable new avenue for research. Further, the development of diurnal models could also be useful in exploring other pathophysiological consequences of circadian disruption, such as the development of insulin resistance, elevated glucose levels and heart hypertrophy (i.e. the ‘circadian syndrome’; Bilu et al., 2019c; Zimmet et al., 2019). Even though we have focused on the rationale and evidence supporting the development of diurnal rodent models, major drawbacks to their use are that they are not available through commercial vendors and there is a lack of genomic resources. Conventional mouse models allow for the genetic engineering of individuals that possess the same mutations found in human populations and the ability to better track the genetics that underlie individual differences in responses to environmental treatments or therapies. Thus, there is a need for investment in whole-genome sequence data and functional annotation to further develop a diurnal rodent model to better understand how genetics influences the effects of light on mood disorders.
